# Supplementary material for: The Evolution and Characterization of the RNA Interference Pathways in Lophotrochozoa
Source: Genome Biol Evol. 2024 May 7;16(5):evae098. doi: 10.1093/gbe/evae098 (PMC11114477; doi:10.1093/gbe/evae098)
Supplement: evae098_Supplementary_Data [file evae098_supplementary_data.zip › Supplementary_materials.zip!/selected_miRNAs/CrGi_selected_miRNAs/CrGi_NC_047564.1_20862.pdf]

Provisional ID	:	NC_047564.1_20862
Score total	:	403.9
Score for star read(s)	:	3.9
Score for read counts	:	397.8
Score for mfe	:	1.2
Score for randfold	:	1.6
Score for cons. seed	:	-0.6
Total read count	:	792
Mature read count	:	659
Loop read count	:	0
Star read count	:	133

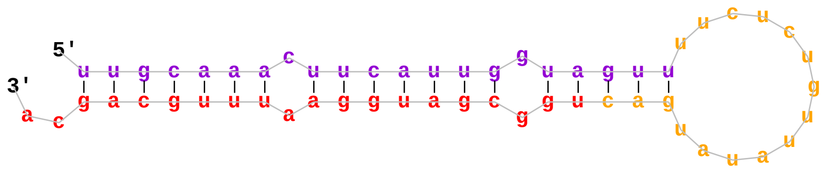

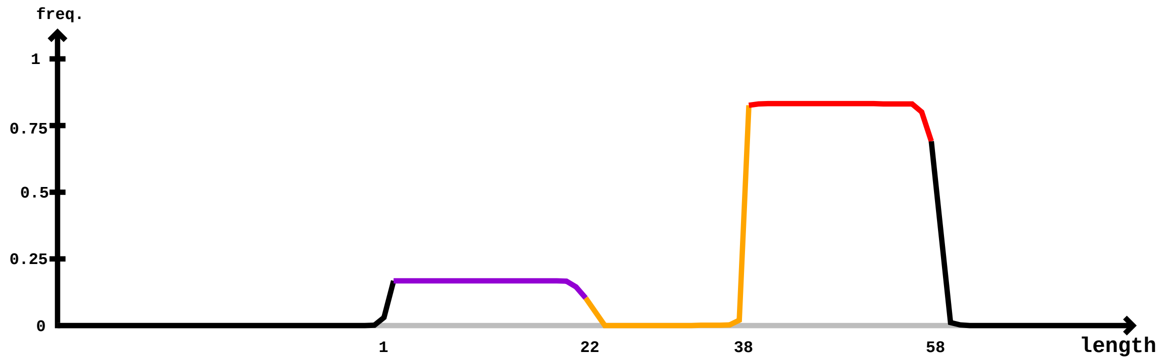


```
                                                                  Star                                  Mature
5'- uuucugugaauuuccuuuaaauuguaauaguuguugcaaacuucauugguaguuuucucuguuauaugacuggcgauggaauuugcagcaaugauugcaauagcaacagc  -3'   obs
    uuucugugaauuuccuuuaaauuguaauaguuguugcaaacuucauugguaguuuucucuguuauaugacuggcgauggaauuugcagcaaugauugcaauagcaacagc         exp
    ....................((((((((.(((((((((((.(((((((.(((((.............))))).))))))).))))))))))).)))))))).........   reads     mm     sample
    ................................guugcaaacuucauugguag..........................................................   3         0      Ov2
    ................................guugcaaacuucauugguagu.........................................................   3         0      Ov2
    .................................uugcaaacuucauugguagu.........................................................   4         0      Ov2
    .................................uugcaaacuucauugguUguu........................................................   1         1      Ov2
    .................................uugcaaacuucauugguaguu........................................................   11        0      Ov2
    .....................................................................cuggcgauggaauuugcagca....................   1         0      Ov2
    .....................................................................cuggcgauggaauuugcagcaa...................   2         0      Ov2
    ......................................................................uggcgauggaauuugcag......................   6         0      Ov2
    ......................................................................uggcgauggaauuugcagc.....................   20        0      Ov2
    ......................................................................uggcgauggaauuugcagca....................   85        0      Ov2
    ......................................................................uggcgauggaauuugcagcaa...................   30        0      Ov2
    ......................................................................uggcgauggaauuugcagcaC...................   1         1      Ov2
    ......................................................................uggcgauggaauuugcagcaaAg.................   1         1      Ov2
    .......................................................................ggcgauggaauuugcagca....................   1         0      Ov2

    ...............................uguugcaaacuucauugguag..........................................................   1         0      Ov1
    ................................guugcaaacuucauuggua...........................................................   1         0      Ov1
    ................................guugcaaacuucauugguag..........................................................   2         0      Ov1
    ................................guugcaaacuucauugAuaguu........................................................   1         1      Ov1
    .................................uugcaaacuucauugguagu.........................................................   6         0      Ov1
    .................................uugcaaacuucauugguaguu........................................................   14        0      Ov1
    .....................................................................cuggcgauggaauuugcagc.....................   1         0      Ov1
    .....................................................................cuggcgauggaauuugcagcaa...................   1         0      Ov1
    ......................................................................uggcgauggaauuugcag......................   6         0      Ov1
    ......................................................................uggcgauggaauuugcagc.....................   19        0      Ov1
    ......................................................................Gggcgauggaauuugcagca....................   1         1      Ov1
    ......................................................................uggcgauggaauuugcagca....................   80        0      Ov1
    ......................................................................uggcgauggaauuugcagcaa...................   14        0      Ov1
    .......................................................................ggcgauggaauuugcagca....................   1         0      Ov1
    ........................................................................gcgauggaauuugcagca....................   1         0      Ov1

    ................................guugcaaacuucauugguag..........................................................   2         0      Mt1
    ................................guugcaaacuucauugguagu.........................................................   3         0      Mt1
```

Star Mature

```
uuucugugaauuuccuuuaaauuguaauaguuguugcaaacuucauugguaguuuucucuguuauaugacuggcgauggaauuugcagcaaugauugcaauagcaacagc
.................................uugcaaacuucauugguag..........................................................   2     0    Mt1
.................................uugcaaacuucauugguagu.........................................................   5     0    Mt1
.................................Gugcaaacuucauugguaguu........................................................   1     1    Mt1
.................................uugcaaacuucauugguaguu........................................................   20    0    Mt1
.................................uugcaaacuucauugguaguuu.......................................................   2     0    Mt1
.................................................................augacuggcgauggaauuu..........................   1     0    Mt1
....................................................................acuggcgauggaauuugcagc.....................   1     0    Mt1
.....................................................................cuggcgauggaauuugcagc.....................   1     0    Mt1
.....................................................................cuggcgauggaauuugcagca....................   3     0    Mt1
.....................................................................cuggcgauggaauuugcagcaa...................   1     0    Mt1
......................................................................uggcgauggaauuugcag......................   4     0    Mt1
......................................................................uggUgauggaauuugcagc.....................   1     1    Mt1
......................................................................uggcgauggaauuugcagc.....................   20    0    Mt1
......................................................................uggcgauggaauuugcagUa....................   1     1    Mt1
......................................................................uggcgauggaauuCgcagca....................   1     1    Mt1
......................................................................uggcgauggaauuugcagca....................   107   0    Mt1
......................................................................uggcgauggaauuugcagcaa...................   39    0    Mt1
......................................................................uggcgauggaauuugcagcaau..................   4     0    Mt1
......................................................................uggcgauggaauuugcagcaaug.................   1     0    Mt1
.......................................................................ggcgauggaauuugcagca....................   1     0    Mt1

................................guugcaaacuucauugguag..........................................................   6     0    Mt2
................................guugcaaacuucauugguagu.........................................................   2     0    Mt2
.................................uugcaaacuucauugguag..........................................................   1     0    Mt2
.................................uugcaaacuucauugguagu.........................................................   10    0    Mt2
.................................uugcaaacuucauugguaCuu........................................................   1     1    Mt2
.................................uugcaaacuucauugguaguu........................................................   30    0    Mt2
.................................uugcaaacuucauugguaguuu.......................................................   1     0    Mt2
.....................................................................cuggcgauggaauuugcagca....................   2     0    Mt2
.....................................................................cuggcgauggaauuugcagcaa...................   2     0    Mt2
......................................................................uggcgauggaauuugcag......................   8     0    Mt2
......................................................................uggcgauggaauuugcagc.....................   23    0    Mt2
......................................................................Cggcgauggaauuugcagc.....................   1     1    Mt2
......................................................................Gggcgauggaauuugcagca....................   1     1    Mt2
......................................................................uggcgauggaauuugcagca....................   125   0    Mt2
......................................................................Cggcgauggaauuugcagca....................   1     1    Mt2
......................................................................uggcgauggaaAuugcagcaa...................   1     1    Mt2
......................................................................uggcgauggaauuugcagcaa...................   34    0    Mt2
......................................................................uggcgauggaauuugcagcaC...................   1     1    Mt2
......................................................................uggcgauggaauuugcagcaau..................   3     0    Mt2
.......................................................................ggcgauggaauuugcagca....................   1     0    Mt2
```
